# 2206. Using Day-Level Registry Data on Patients in OPAT to Assess Heterogeneous Patterns of Time-Varying Antimicrobial Exposure and Unplanned Stopping across Multiple Infection Diagnoses, 2015-2022

**DOI:** 10.1093/ofid/ofad500.1828

**Published:** 2023-11-27

**Authors:** Madison Ponder, Renae Boerneke, Asher J Schranz, Michael Swartwood, Claire E Farel, Alan C Kinlaw

**Affiliations:** University of North Carolina at Chapel Hill, Chapel Hill, NC; UNC Health, Chapel Hill, North Carolina; University of North Carolina, Chapel Hill, NC; University of North Carolina Medical Center, Chapel Hill, North Carolina; UNC Chapel Hill, Chapel Hill, North Carolina; University of North Carolina School of Pharmacy, Chapel Hill, North Carolina

## Abstract

**Background:**

Patients receiving outpatient parenteral antimicrobial therapy (OPAT) are often medically complex and require carefully tailored treatments to address severe and often concomitant infections.

**Methods:**

We conducted a cohort study of patients enrolled in the University of North Carolina Medical Center OPAT program between March 2015 and December 2022 (2358 OPAT courses among 2072 unique patients). Patients were followed from OPAT regimen initiation (day 0) until observed treatment stop date, based on a grace period of 5 days to confirm discontinuation. Using hierarchical and mutually exclusive categorization schema, we classified patients into 1 of 10 infection diagnosis groups (Figure 1A), and classified all observed antibiotic cocktails (64 distinct antimicrobial medications comprising 520 distinct cocktails) into 18 treatments (Figure 1B). For each patient, we assessed their planned OPAT duration to classify their observed discontinuation as “planned,” “unanticipated early stop,” or “unanticipated prolonged treatment.” Stratified by infection diagnosis and treatment classifications, we used stacked bar charts to describe treatment heterogeneity, and Aalen-Johansen-based cumulative incidence curves to estimate occurrence of each type of discontinuation amid competing events.

**Results:**

The cohort was 59.7% male with median age 56 years (IQR 45-66); the most common infection diagnosis was osteomyelitis +/- diabetic foot infection (28.1%, Figure 1A). Medication exposures were heterogeneous within and across infection diagnoses (Figure 1A and 1B). Median planned OPAT duration was 36 days (middle 90% 15-44). Overall cumulative incidence of unanticipated early discontinuation was 11.9%; overall incidence of unanticipated prolonged treatment was 22.9% (Figure 2).

**Figure:**
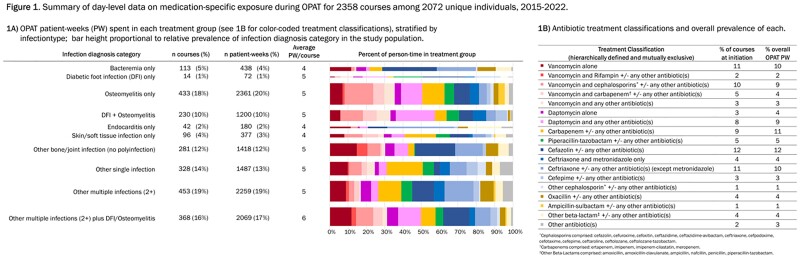

**Figure:**
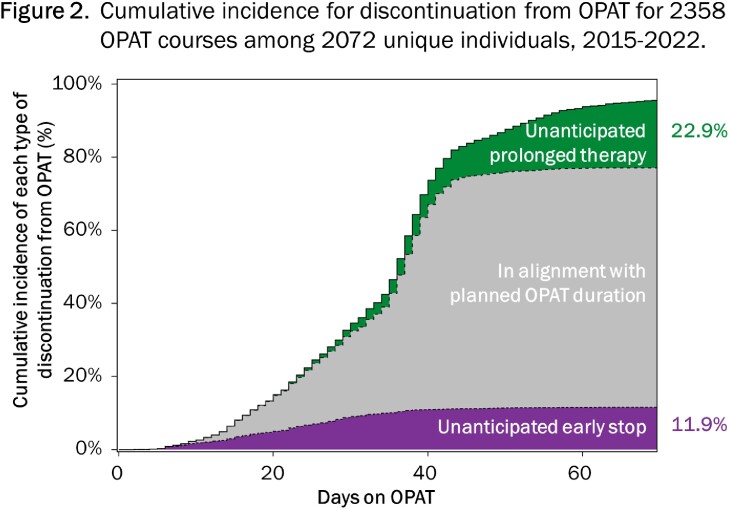

**Conclusion:**

Day-level data on medication use in this registry provided evidence of heterogeneity in medication exposure and unplanned discontinuation from OPAT, within and across infection diagnoses. These data highlight the need for multi-layered ascertainment of medication exposure, microorganisms, and infection diagnosis in this medically complex patient population to inform surveillance for adverse effects and comparative effectiveness research for post-discharge antibiotic treatment.

**Disclosures:**

**Asher J. Schranz, MD, MPH**, WoltersKluwer: Honoraria **Alan C. Kinlaw, PhD, MSPH**, Genentech: Grant/Research Support

